# Supplementary figures and images for: Visual physiology of the layer 4 cortical circuit in silico
Source: PLoS Comput Biol. 2018 Nov 12;14(11):e1006535. doi: 10.1371/journal.pcbi.1006535 (PMC6258373; doi:10.1371/journal.pcbi.1006535)

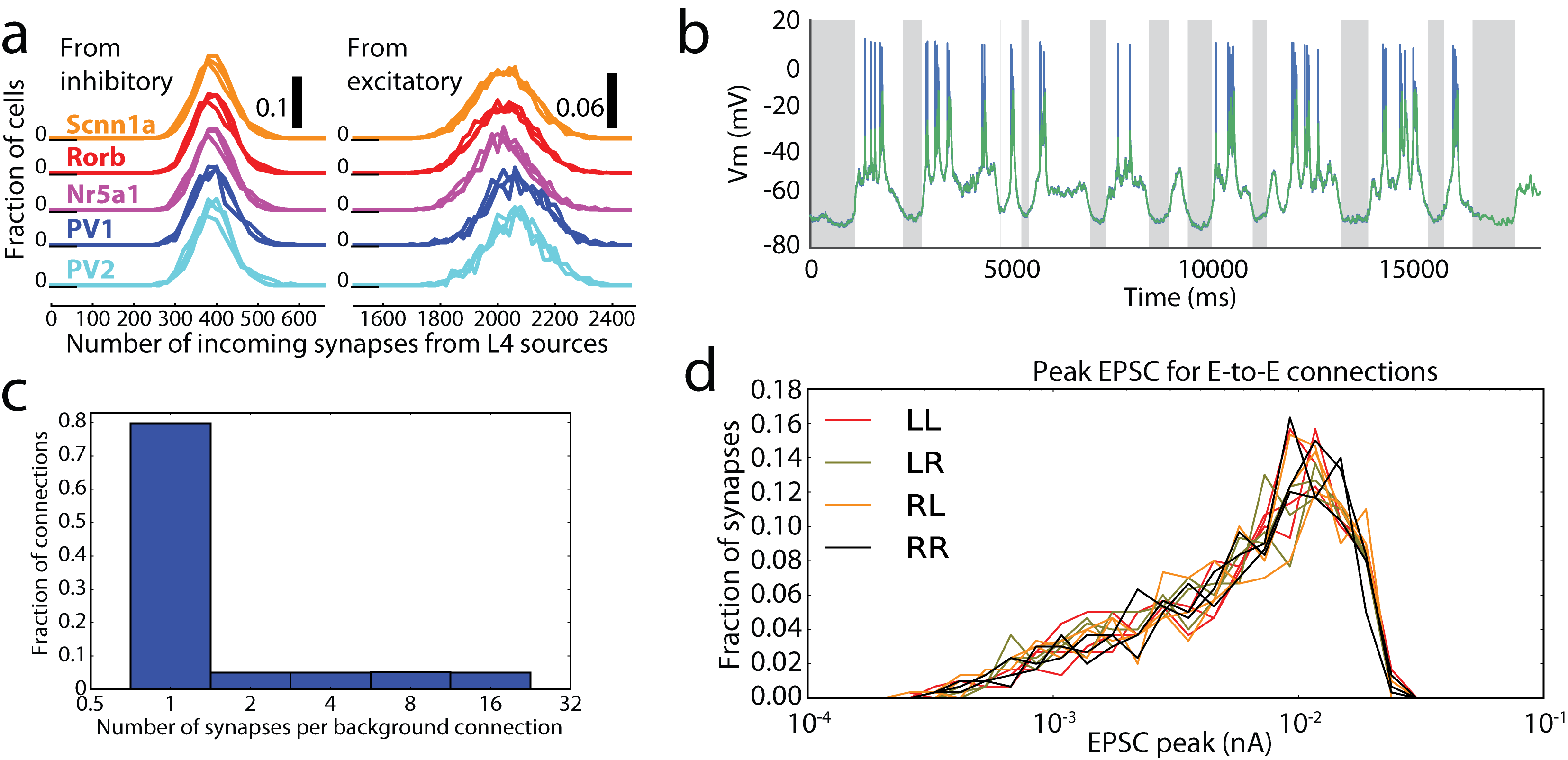

Supplement: S1 Fig — (a) Distribution of the number of synapses from L4 sources onto the five cell types in the network model. Three curves are shown for each type, corresponding to three independent model instantiations. (b) An example trace of the membrane voltage Vm from patch clamp recordings from V1 L2/3 cells in vivo (under anesthesia) during spontaneous activity. Blue portion of the trace corresponds to the data points removed from the analysis of the state characteristics (clipped spikes). The gray stripes mark the identified rest states. (c) Number of synapses formed on an L4 cell by a connection from a single background source. The distribution of synapse numbers over all background connections is shown, combined from the three independent models. (d) Distribution of peak somatic EPSC values for E-to-E recurrent connections in all simulated models (for biophysical neurons), on the log scale. Data for three models per system type (LL, LR, RL, or RR) are shown (see Main Text). (TIF) [file pcbi.1006535.s001.tif]

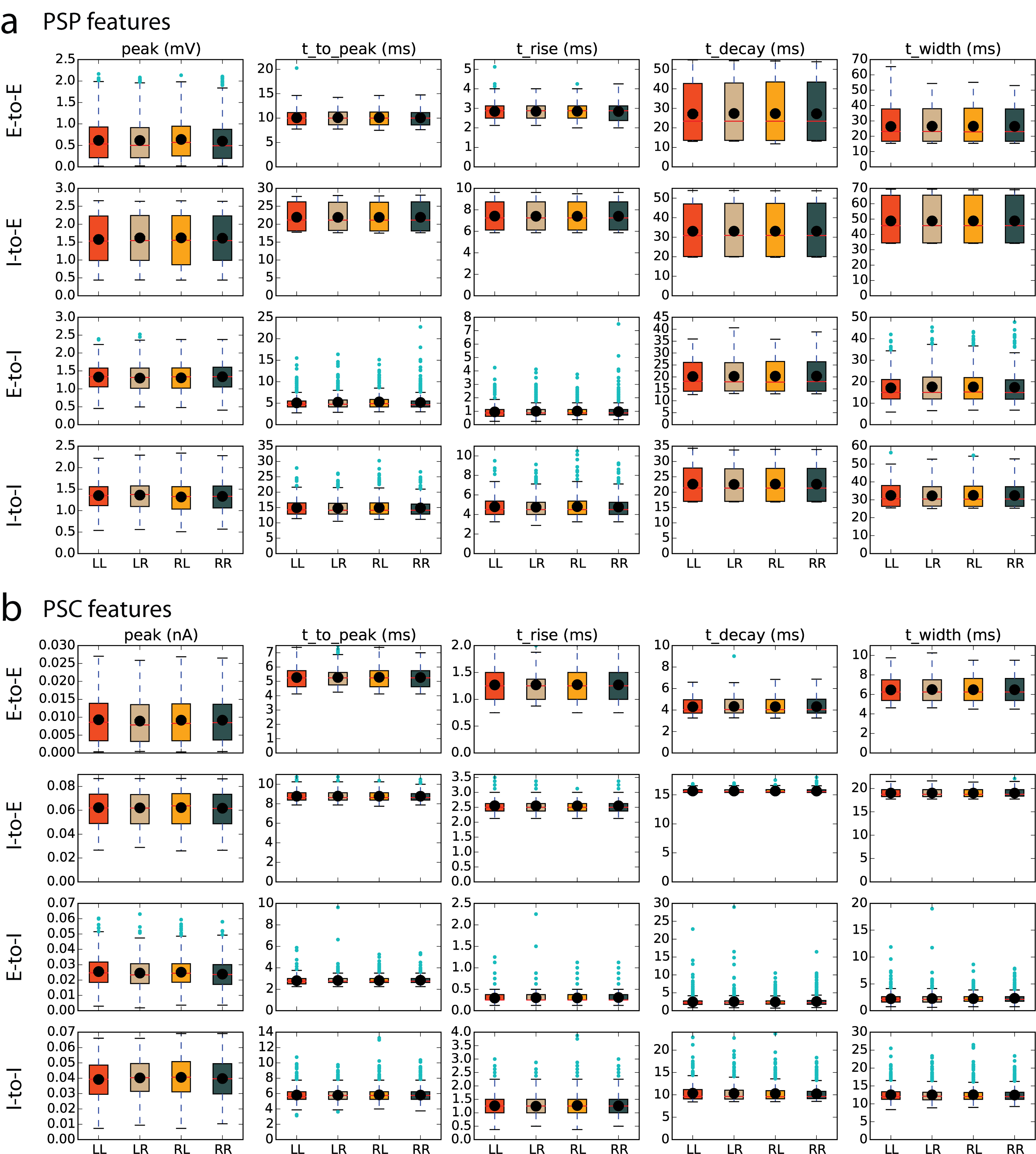

Supplement: S2 Fig — See Online Methods for details of box plots. The features are voltage or current peak (“peak”), time from spike to peak (“t_to_peak”), rise time (“t_rise”), decay time (“t_decay”) and the PSP or PSC width (“t_width”). (a) Somatic PSP features. (b) Somatic PSC features. For each feature and each model type (i.e., LL, LR, RL, or RR), the sample sizes are n = 900 for “E-to-E” and “I-to-E” and n = 600 for “E-to-I” and “I-to-I”. (TIF) [file pcbi.1006535.s002.tif]

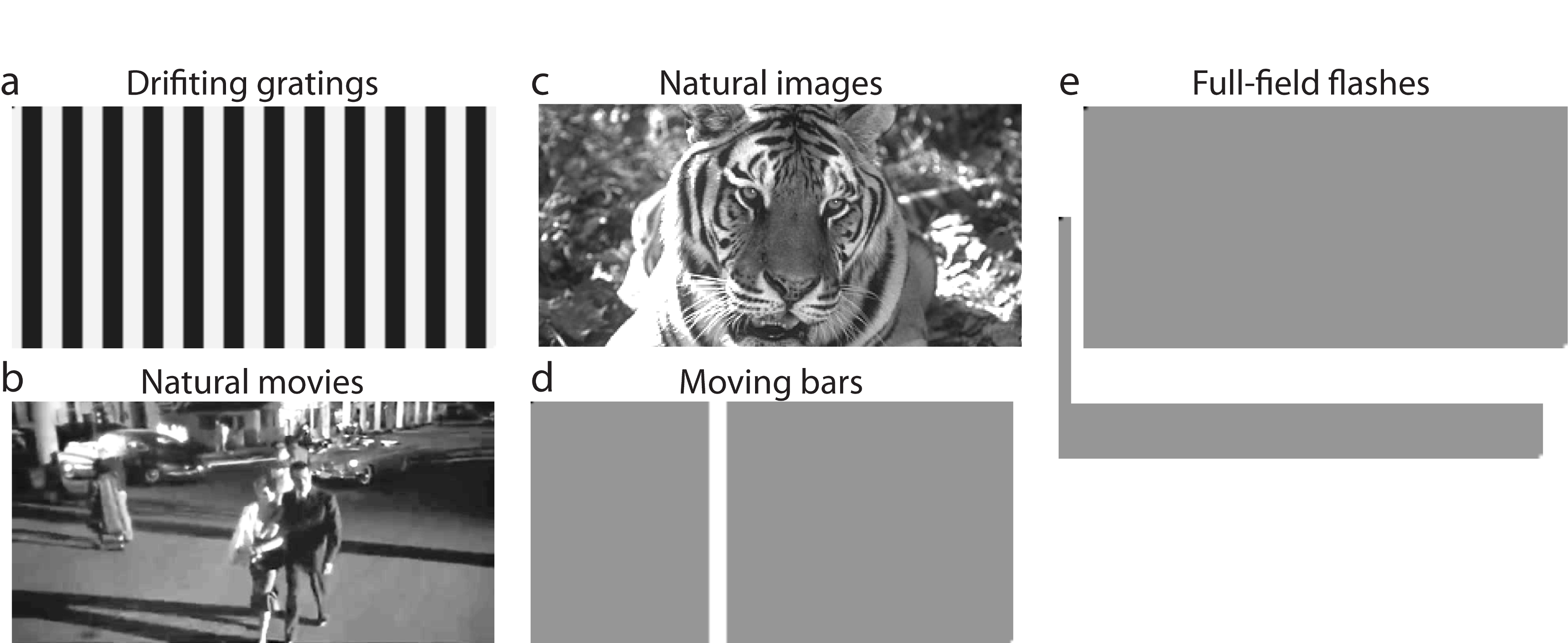

Supplement: S3 Fig — Examples of visual stimuli used for simulations and experiments are shown, such as (a) drifting gratings, (b) natural movies, (c) static natural images, (d) moving white or black bars, and (e) full-field flashes. (TIF) [file pcbi.1006535.s003.tif]

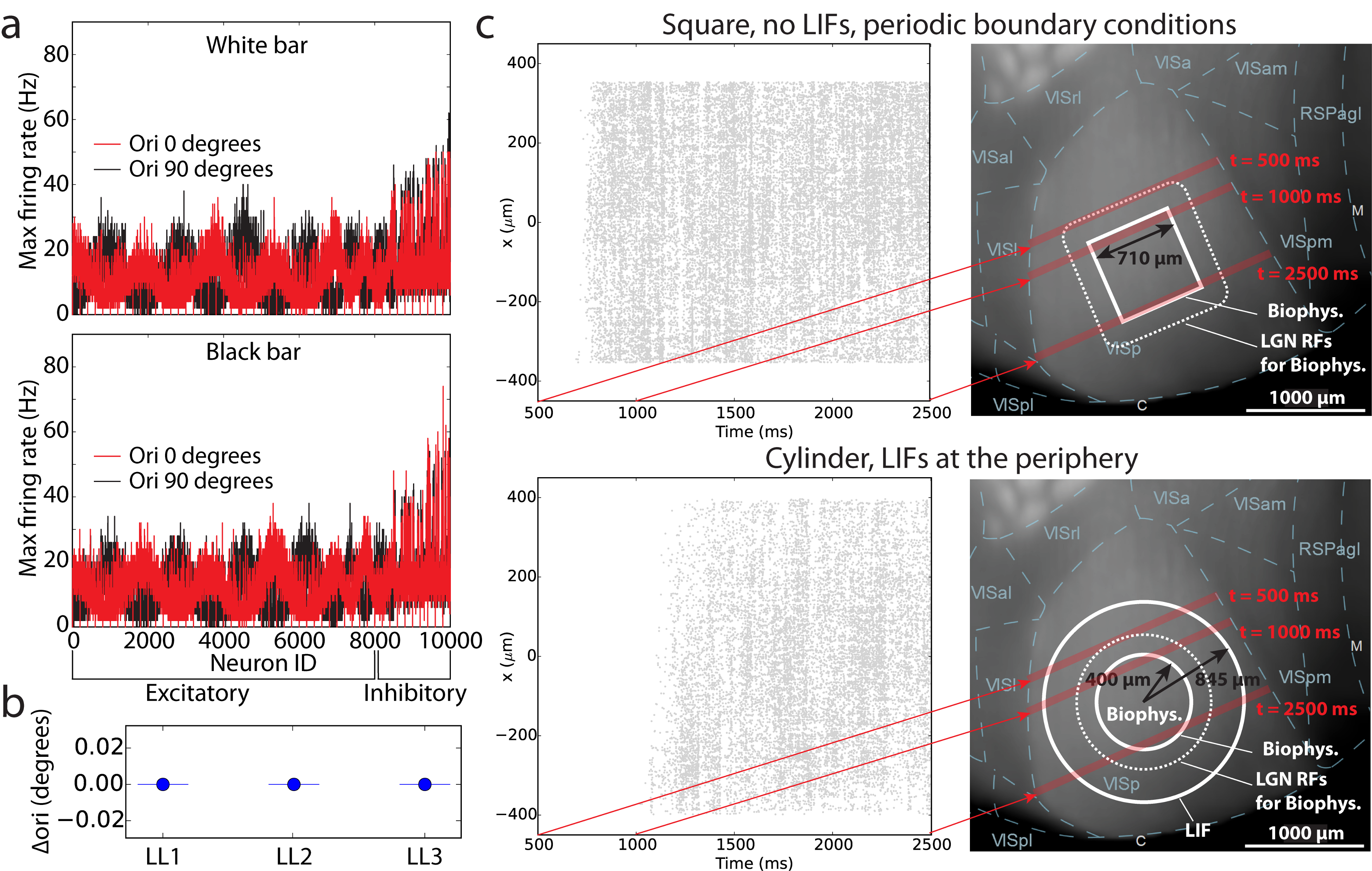

Supplement: S4 Fig — (a) Responses of each biophysical neuron in one model to black and white bars; either a vertical bar was moving in a horizontal direction (“Ori 0 degrees”) or a horizontal bar was moving in a vertical direction (“Ori 90 degrees”). The responses shown were obtained from time-dependent firing rates (in 50 ms bins) averaged over all trials of a given stimulus; the maximum over all bins is computed for each neuron. The neuron IDs for each type are arranged according to the neurons’ assumed direction preference for gratings (see Online Methods), from 0 degrees for the first ID of a type to 360 degrees for the last (hence the pseudo-periodicity apparent in the plots). The types are Scnn1a (IDs 0 to 3699), Rorb (3700 to 6999), Nr5a1 (7000 to 8499), PV1 (8500 to 9299), and PV2 (9300 to 9999). (b) The difference ΔOri between the preferred orientations of a neuron according to responses to gratings and to bars, averaged over all excitatory neurons that prefer 0, 90, 180, or 270 degrees for gratings. The averages and standard deviations are exactly zero for all three models tested. (c) Spike rasters (left) for biophysical neurons from pilot simulations of responses to a horizontally moving white bar, using different model layouts illustrated on the right. For each spike, the position of the neuron along the x dimension (which coincides with the direction of the moving bar) is plotted versus spike time. Top, a model without LIF neurons, with biophysical neurons confined to a rectangular area, and using periodic boundary conditions for connectivity. Bottom, a model with biophysical neurons confined to a cylinder, with LIF neurons distributed at the periphery (no periodic boundary conditions)–that is, the model layout chosen for all simulations reported in the Main Text. The approximate extent of the receptive fields (RFs) of LGN cells that feed into the biophysical portion of the model are marked by white dashed lines. Note that in these preliminary test simulations, the paramet [file pcbi.1006535.s004.tif]

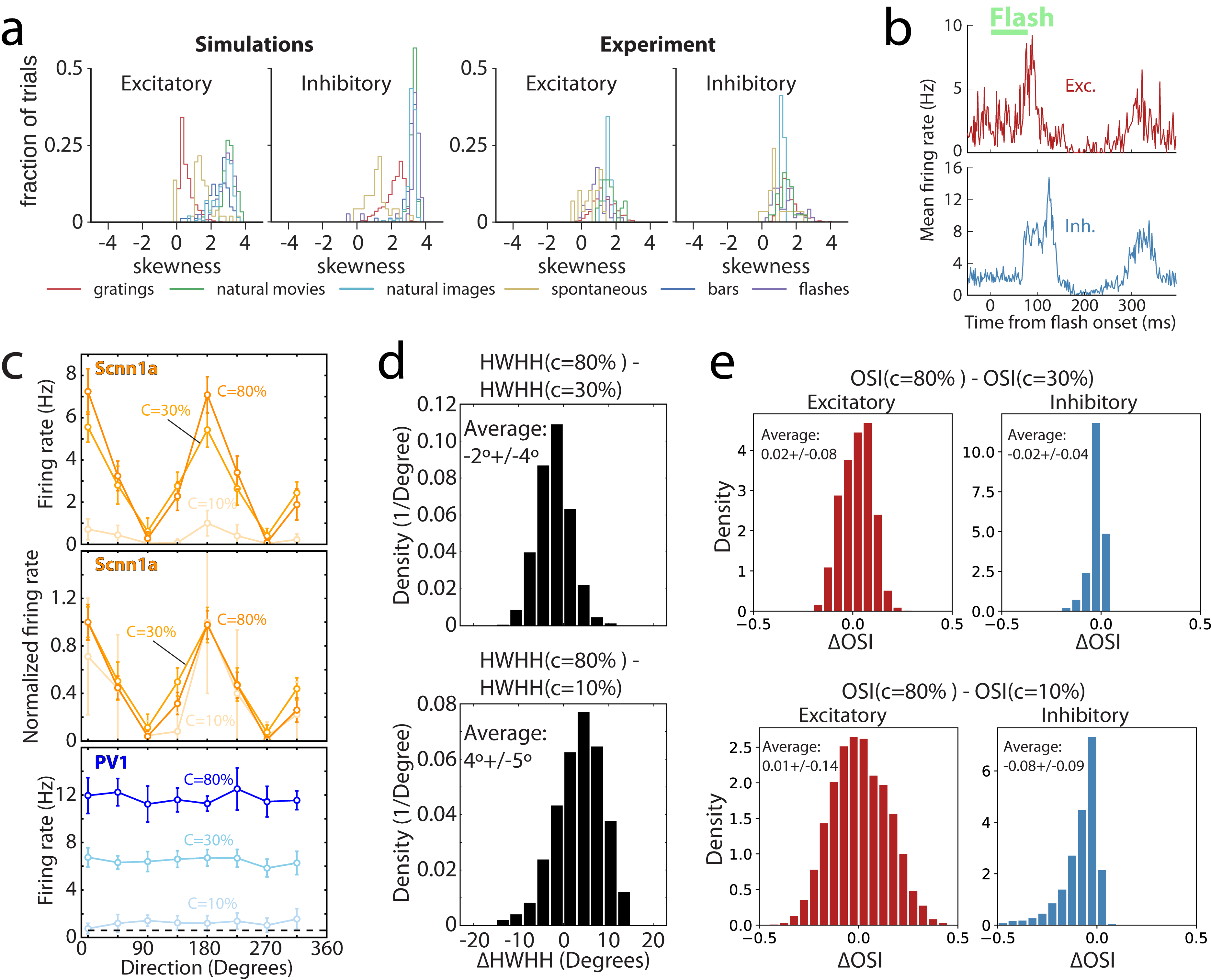

Supplement: S5 Fig — (a) Distributions of skewness of firing rates. Left, simulation; right, electrophysiological experimental recordings. (b) The PSTHs from experimental electrophysiological recordings in response to a 50 ms flash (average over all L4 excitatory cells or all inhibitory cells recorded, and all trials, in 2 ms bins). (c) Example tuning curves of a single Scnn1a or PV1 cell to drifting gratings at contrasts C = 80% and C = 10%. For the Scnn1a cell, responses normalized to the peak of the tuning curve are also shown (middle). The data are averages over 10 trials. Error bars: standard deviation. Dashed lines: spontaneous rate (it is close to zero for the example Scnn1a cell shown). (d) Summary of responses to the gratings at different contrasts (C = 30% or 10% vs. C = 80%). The distributions of differences ΔHWHH = HWHH(C = 80%)—HWHH(C = 30%) (top) and ΔHWHH = HWHH(C = 80%)—HWHH(C = 10%) (bottom), are shown for all excitatory cells, with the average +/- standard deviation indicated. (e) Same as (d) for the differences of OSI for excitatory (red) and inhibitory (blue) neurons. (TIF) [file pcbi.1006535.s005.tif]

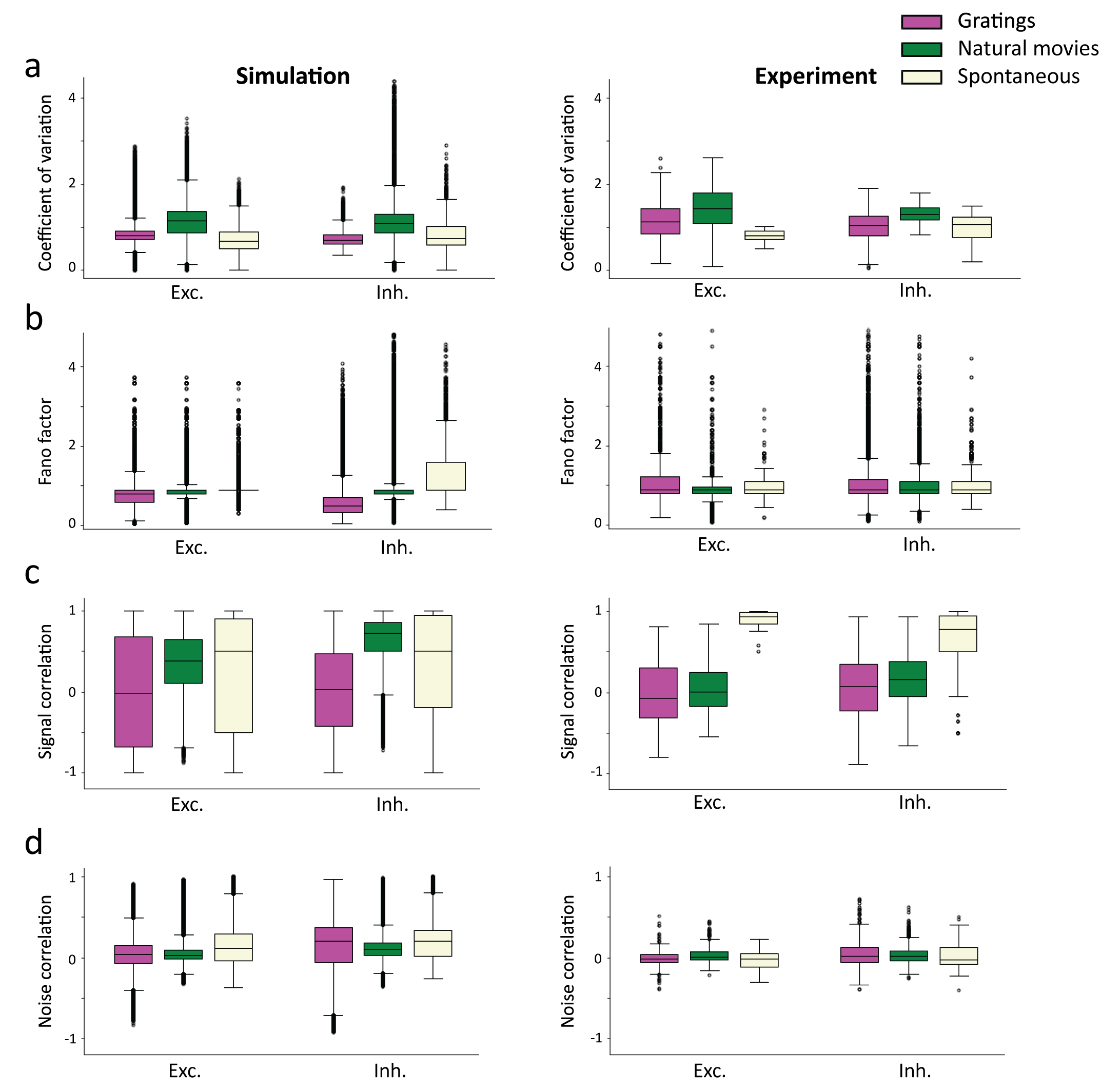

Supplement: S6 Fig — Comparison of variability and correlations between simulations (left) and experiment (right). Results of the analysis are shown for gratings (magenta), natural movies (green) and spontaneous activity (beige). (a) Coefficient of variation of inter-spike intervals. (b) Fano factor. (c) Signal correlations. (d) Noise correlations. (TIF) [file pcbi.1006535.s006.tif]

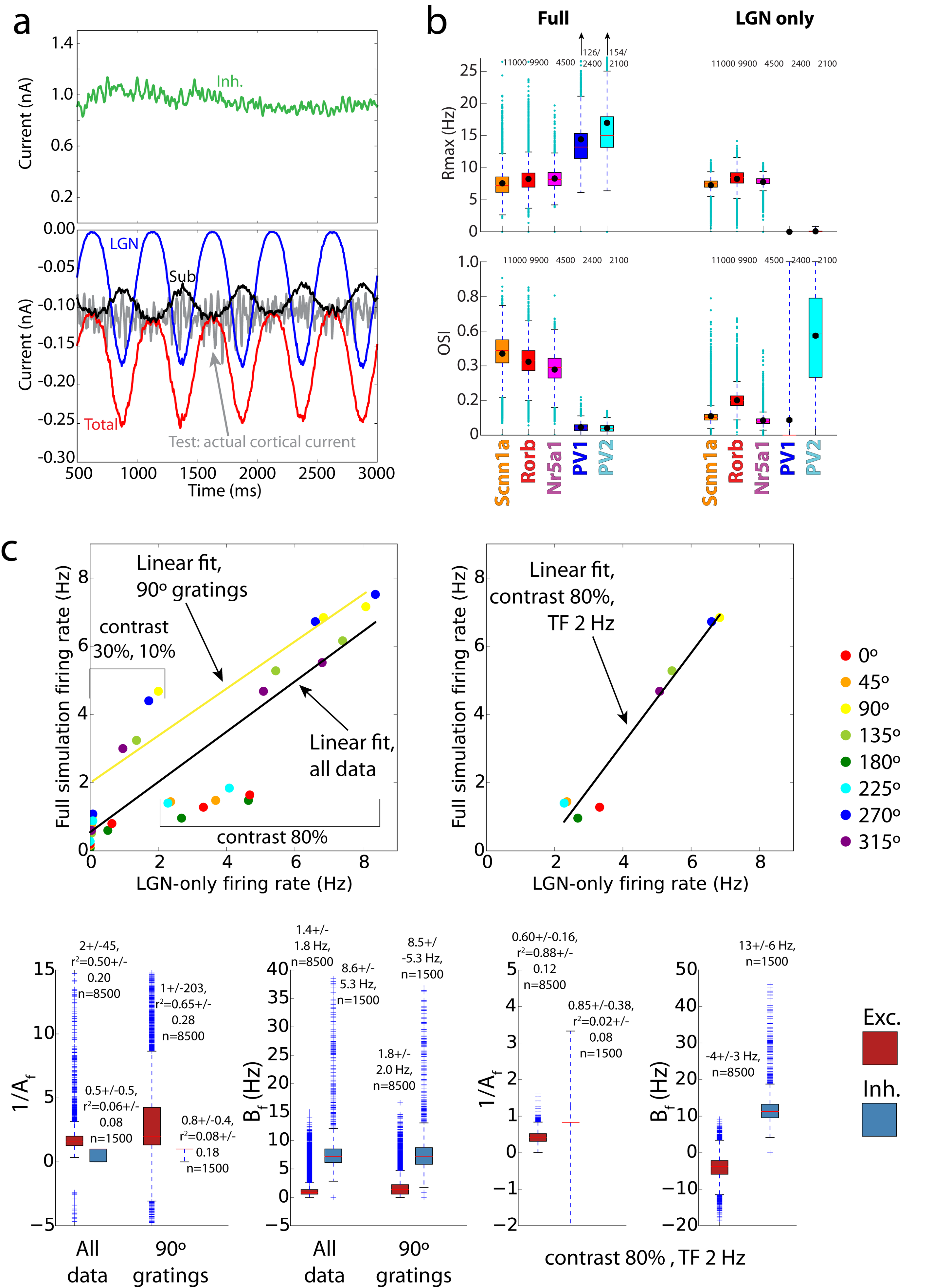

Supplement: S7 Fig — (a) Temporal dynamics of current amplification (for a TF = 2 Hz grating). The total and LGN-only currents are shown, along with their difference (“Sub”, i.e., the cortical component); the top plot shows the inhibitory current. Data for individual cells for the preferred direction are averaged over 10 trials; the traces are then shifted in time (periodically with respect to the trial start and end, i.e., 500 ms and 3000 ms) so that the phases of the LGN component are aligned across all cells. The data are then averaged over all biophysical excitatory cells. Additional tests were performed, where LGN and background inputs to the voltage-clamped cells were eliminated (gray). In this case, the measured current is from the L4 circuit only. This demonstrates that in simulations the oscillation of the “Sub” component (black) is due to a space clamp artifact. (b) Comparison of the maximal responses to drifting gratings (Rmax) and OSI between the full network simulations (“Full”) and the purely feedforward simulations with the inputs to L4 coming only from the LGN filters (“LGN only”). Note that the OSIs of PV1 and PV2 cells are nominally high for the feedforward case because the firing rates of these cells in that situation are very close to zero. An occasional rare spike results in a “strong” response in comparison with zero responses for most trials of most orientations, which leads to elevated OSI values. (c) Comparison of the firing rates in the full network vs. firing rates in a purely feedforward model receiving only inputs from the LGN. Top, an individual Rorb cell (each point is an average over time and over 10 trials). Linear fits are shown for data aggregated from all grating directions, TFs, and contrasts (black), for one selected direction (yellow), and for a fixed contrast and TF (i.e., representing a sample direction tuning curve; right plot). Bottom, summary of linear fits across all cells analyzed. (TIF) [file pcbi.1006535.s007.tif]

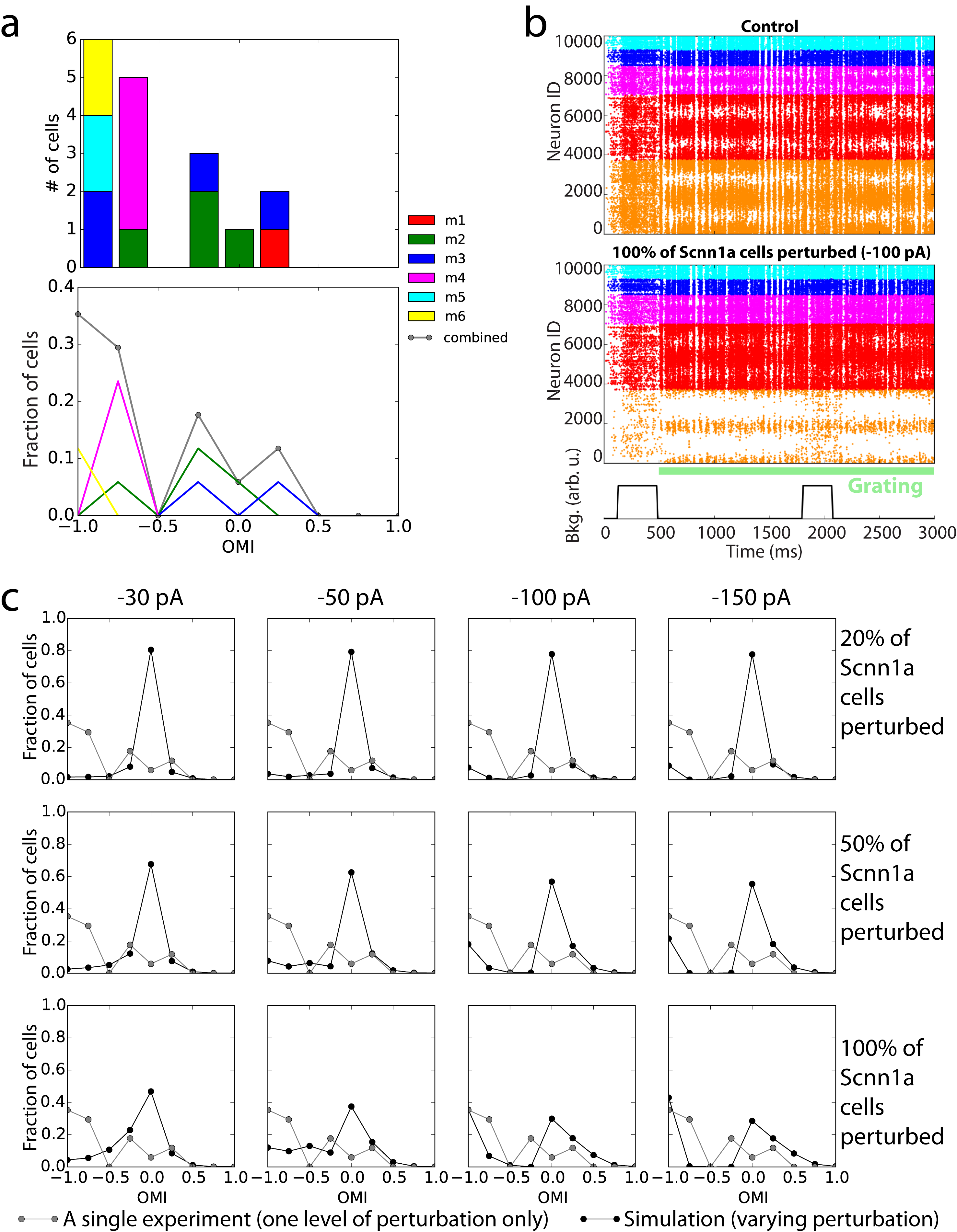

Supplement: S8 Fig — (a) Summary of experimental manipulations. The OMI index data are shown for each cell in all 6 experiments (colors, m1 to m6) in the top plot. The bottom plot shows the same data normalized to the total number of cells from all 6 experiments and the combined normalized distribution (gray). Note that in the bottom plot, the curves for m1, m3, m5, and m6 fully or partially overlap. (b) Spike rasters from two simulations–with and without optogenetic perturbation of the Scnn1a population. In both cases, the same drifting grating is presented (TF = 4 Hz), with the stimulation illustrated at the bottom. The hyperpolarizing current at the level of -100 pA, representing the optogenetic perturbation, was injected in the somata of 100% of the Scnn1a cells. (c) OMI computed across all recorded layer 4 excitatory neurons in experiments and simulations. The experimental curve (gray; it is the same in all plots and same as in (a)) is used as a benchmark. The simulation conditions differ between the plots (in the amount of current injected and the proportion of cells that receive the injected current), and thus the simulation curves (black) are different. (TIF) [file pcbi.1006535.s008.tif]

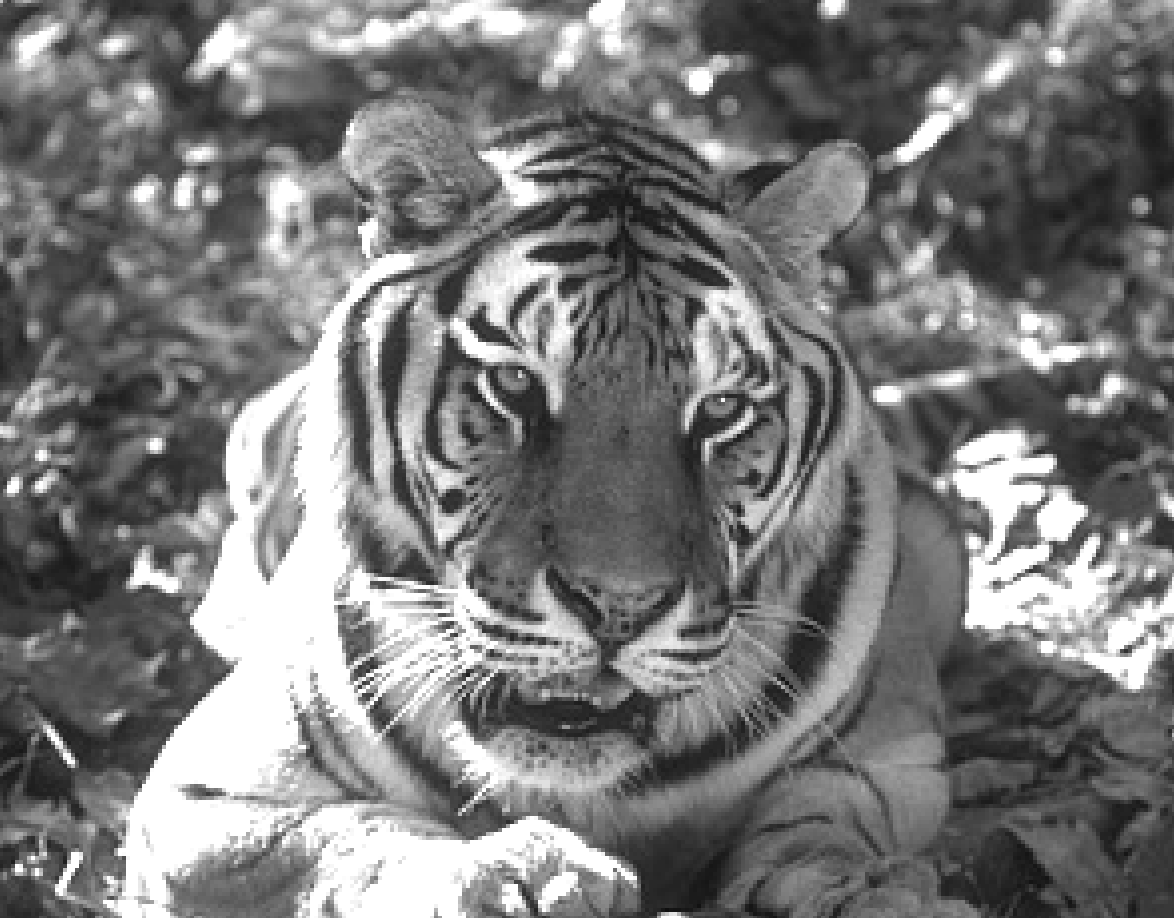

Supplement: SI 4 — (ZIP) [file pcbi.1006535.s014.zip › SI_4/natural_images/img011_BR.tiff]

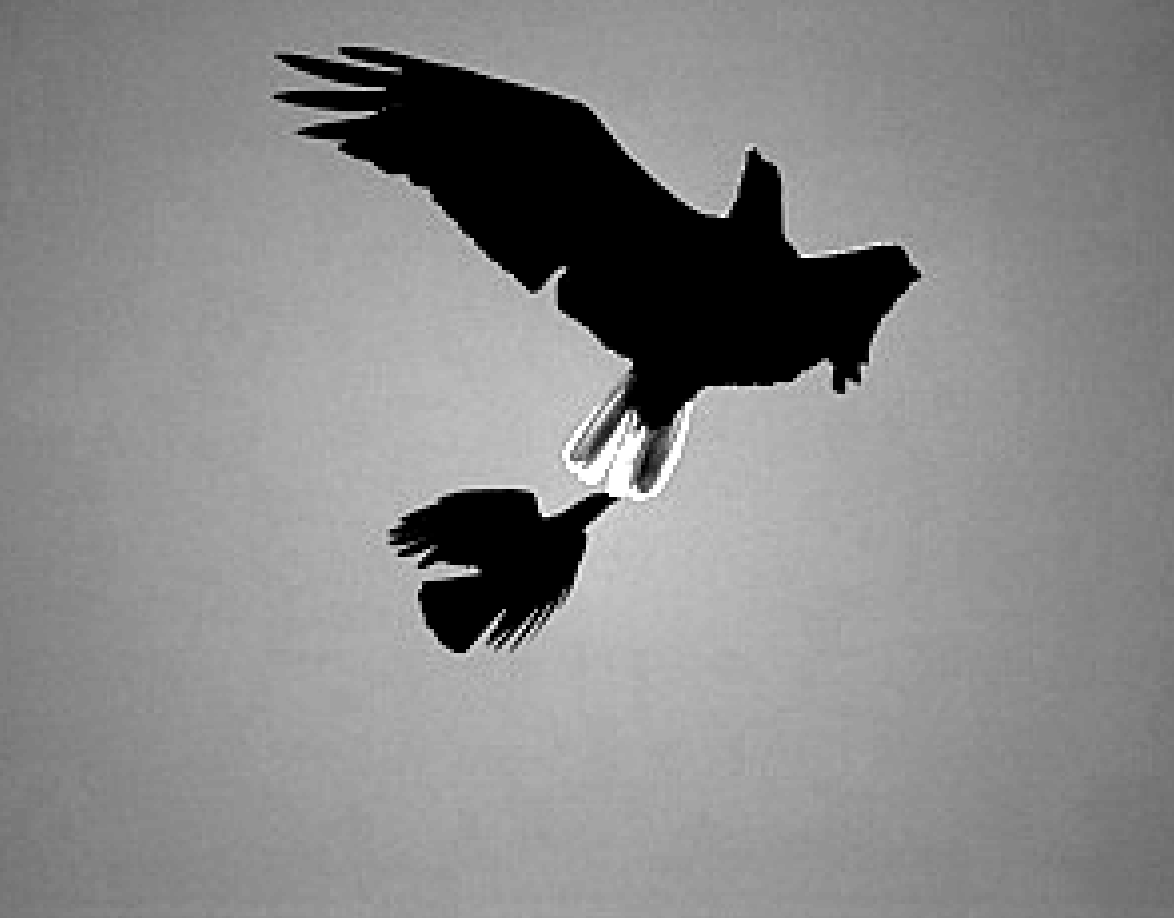

Supplement: SI 4 — (ZIP) [file pcbi.1006535.s014.zip › SI_4/natural_images/img019_BT.tiff]

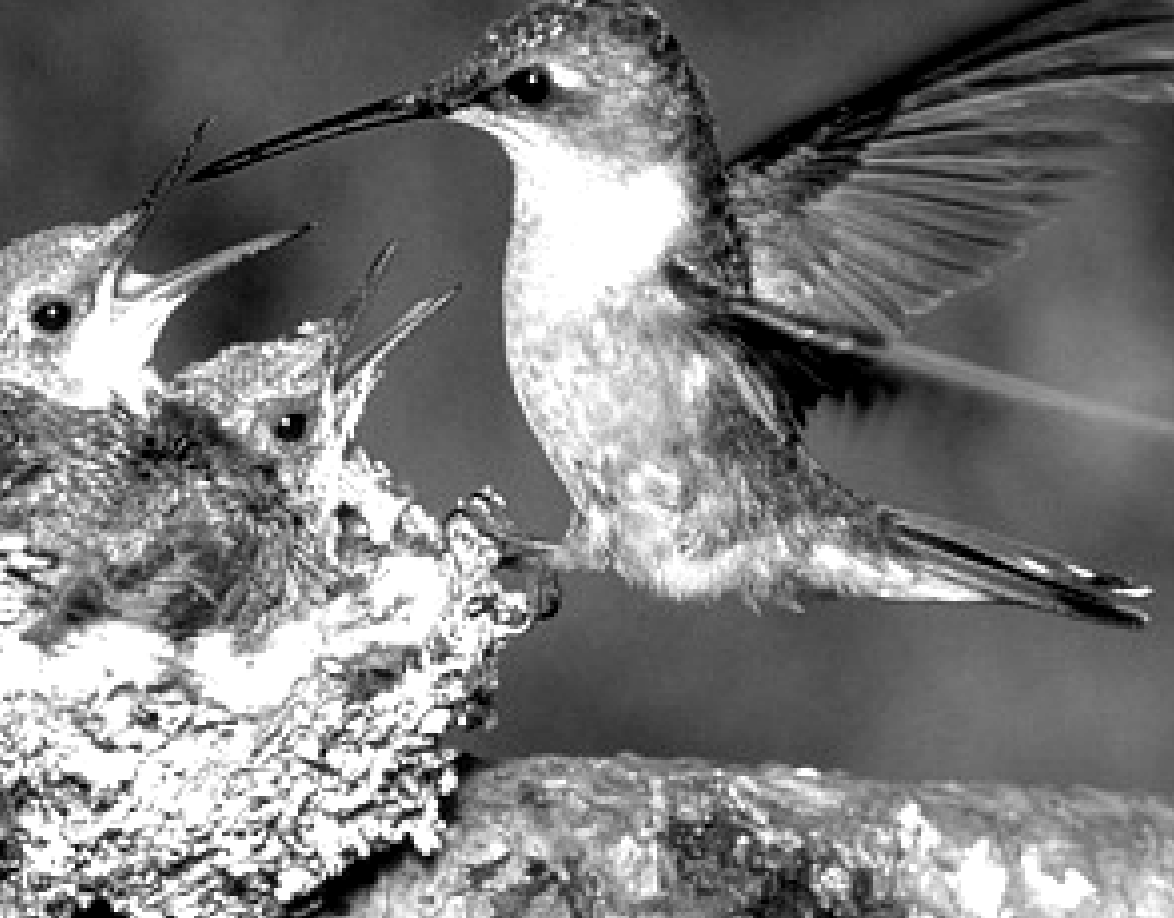

Supplement: SI 4 — (ZIP) [file pcbi.1006535.s014.zip › SI_4/natural_images/img024_BT.tiff]

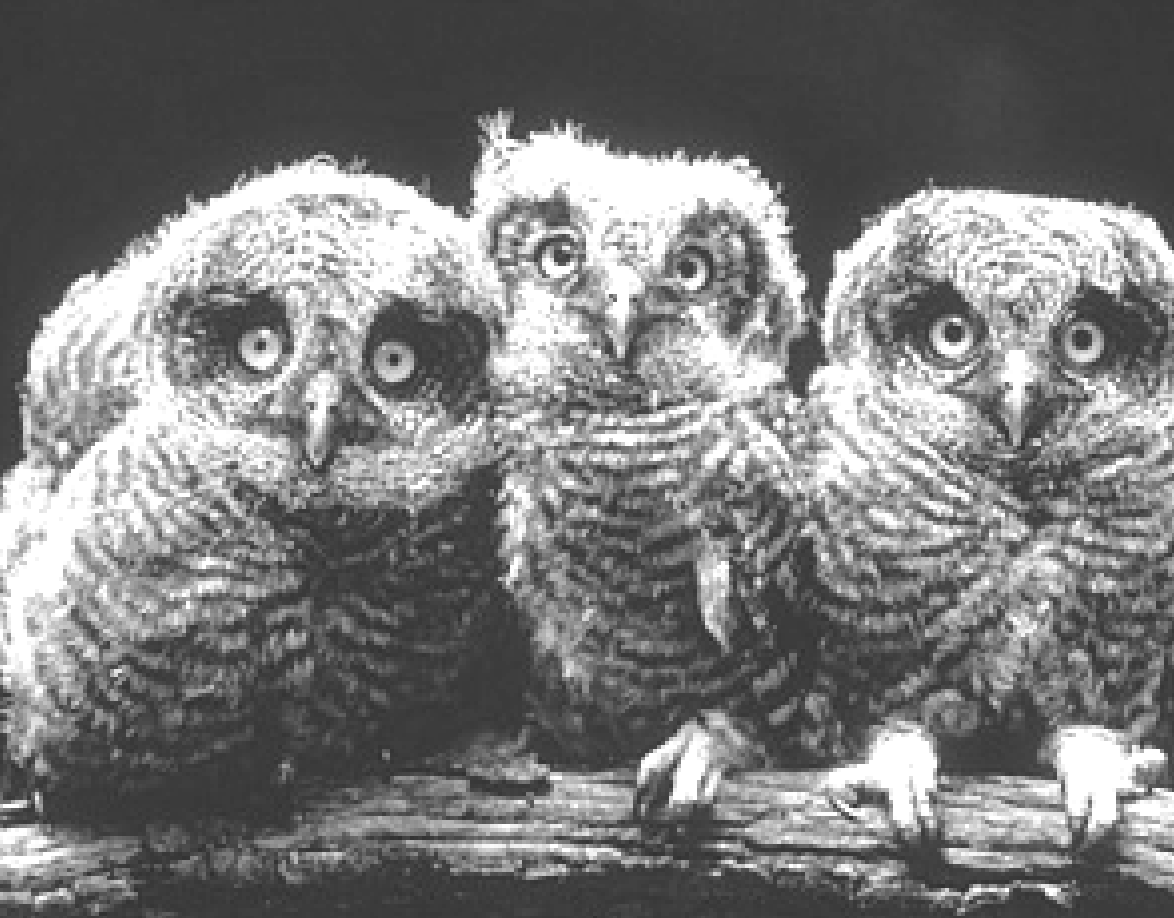

Supplement: SI 4 — (ZIP) [file pcbi.1006535.s014.zip › SI_4/natural_images/img049_BT.tiff]

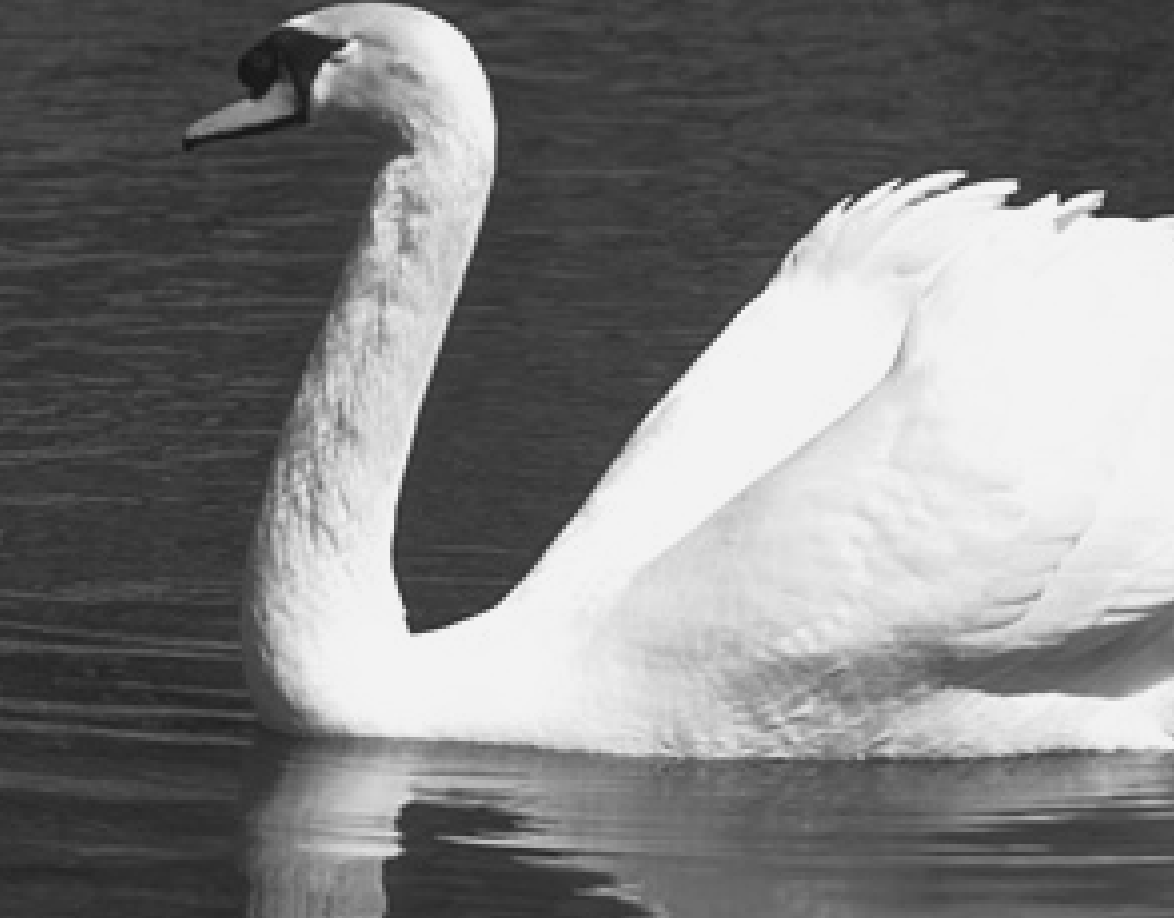

Supplement: SI 4 — (ZIP) [file pcbi.1006535.s014.zip › SI_4/natural_images/img057_BR.tiff]

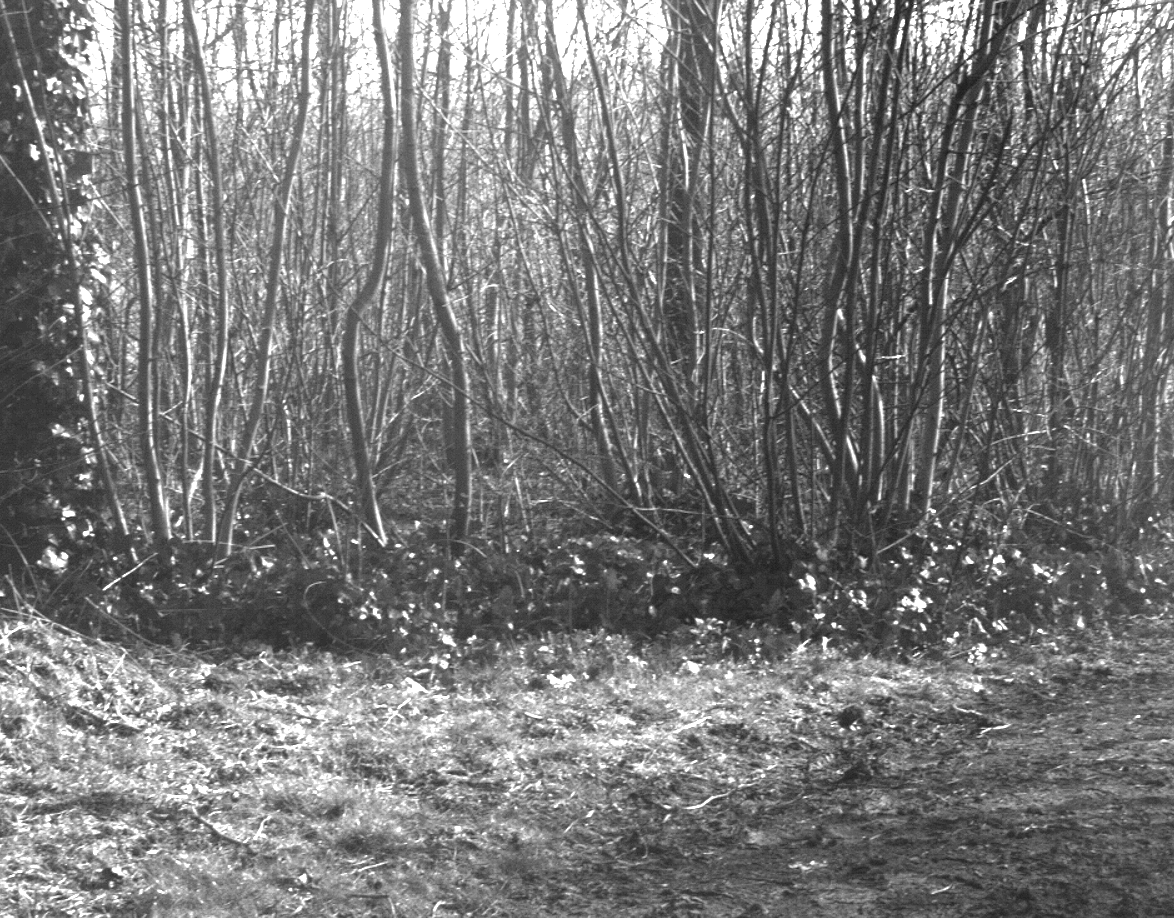

Supplement: SI 4 — (ZIP) [file pcbi.1006535.s014.zip › SI_4/natural_images/img062_VH.tiff]

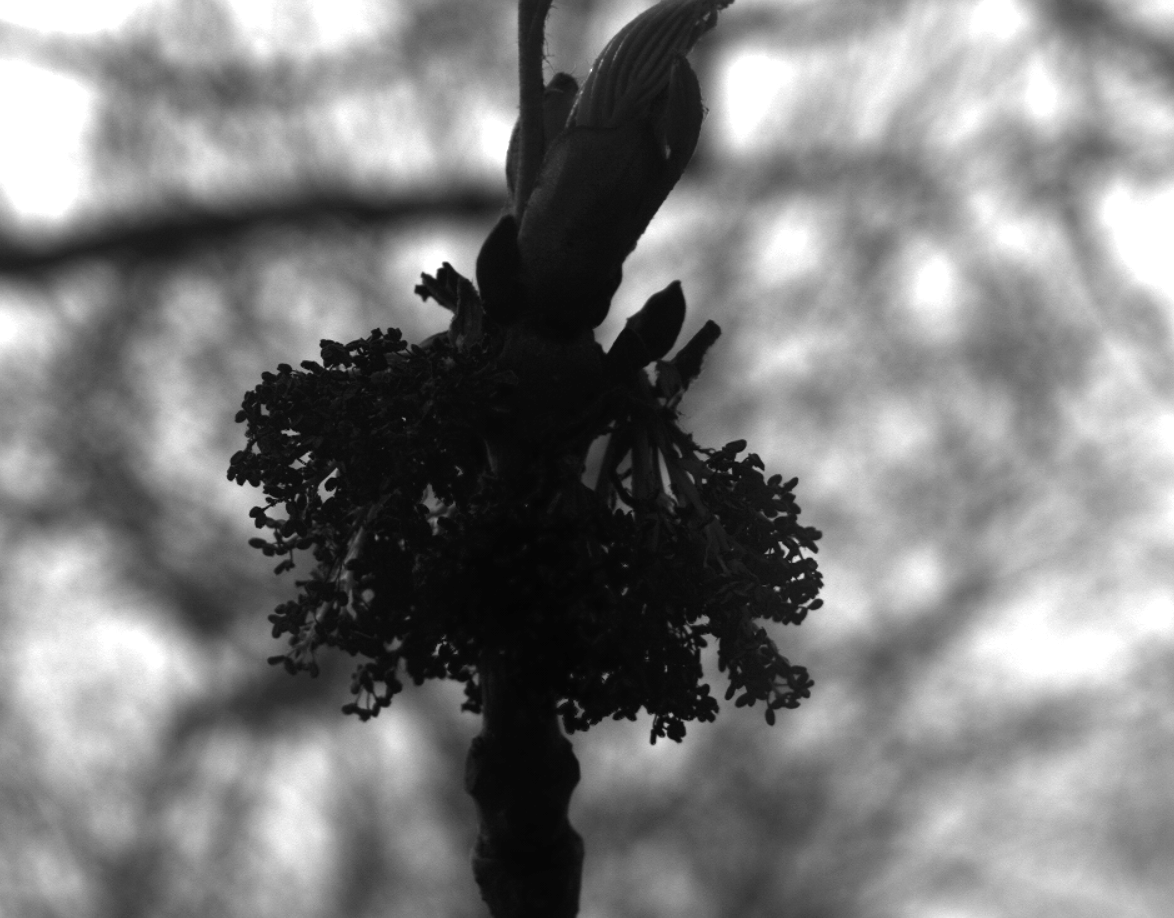

Supplement: SI 4 — (ZIP) [file pcbi.1006535.s014.zip › SI_4/natural_images/img069_VH.tiff]

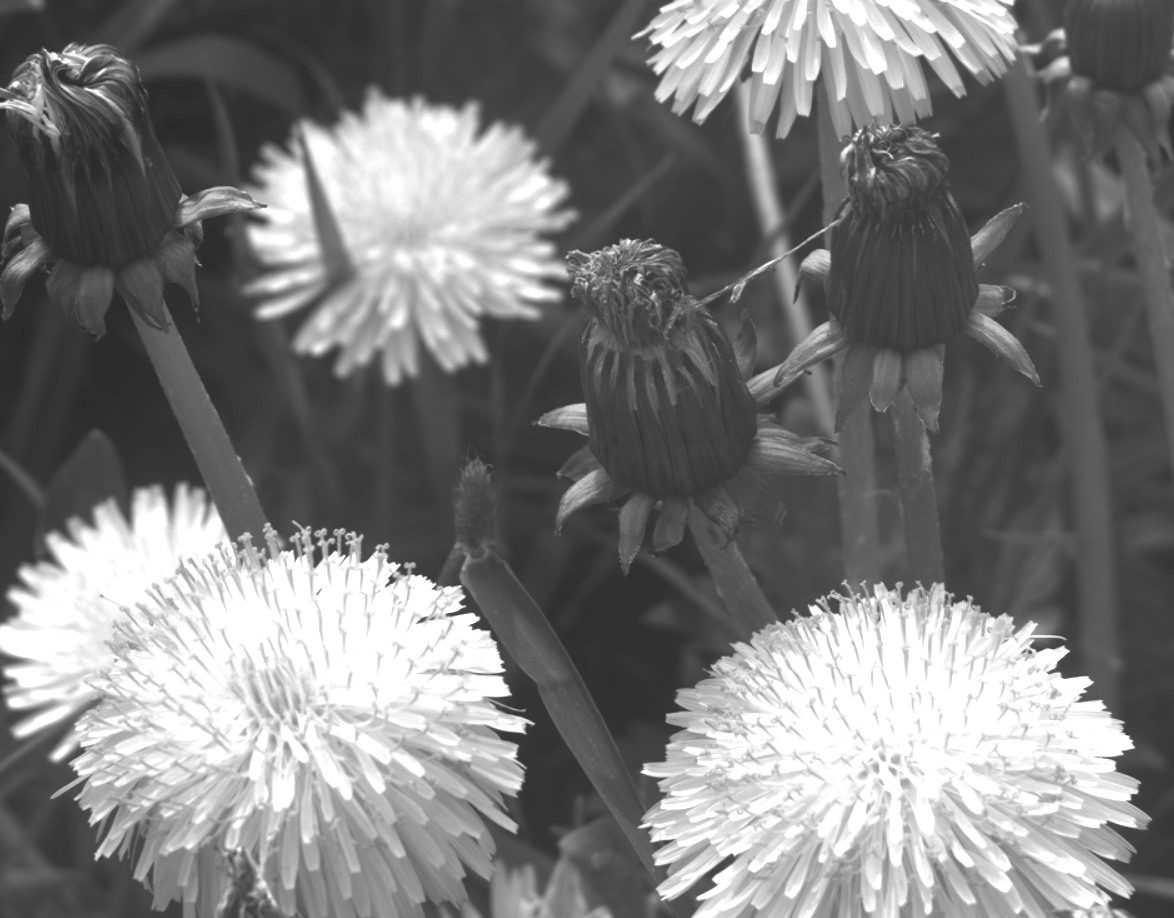

Supplement: SI 4 — (ZIP) [file pcbi.1006535.s014.zip › SI_4/natural_images/img071_VH.tiff]

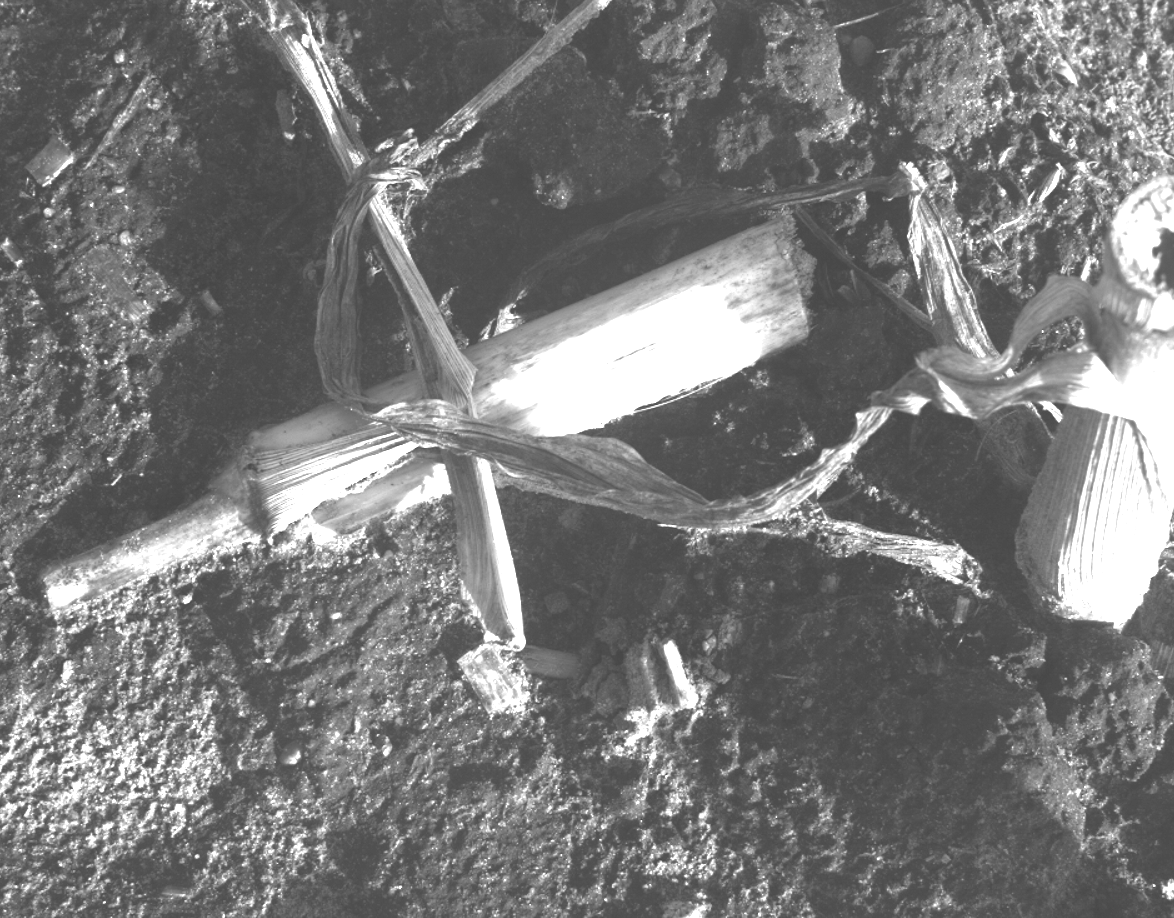

Supplement: SI 4 — (ZIP) [file pcbi.1006535.s014.zip › SI_4/natural_images/img090_VH.tiff]

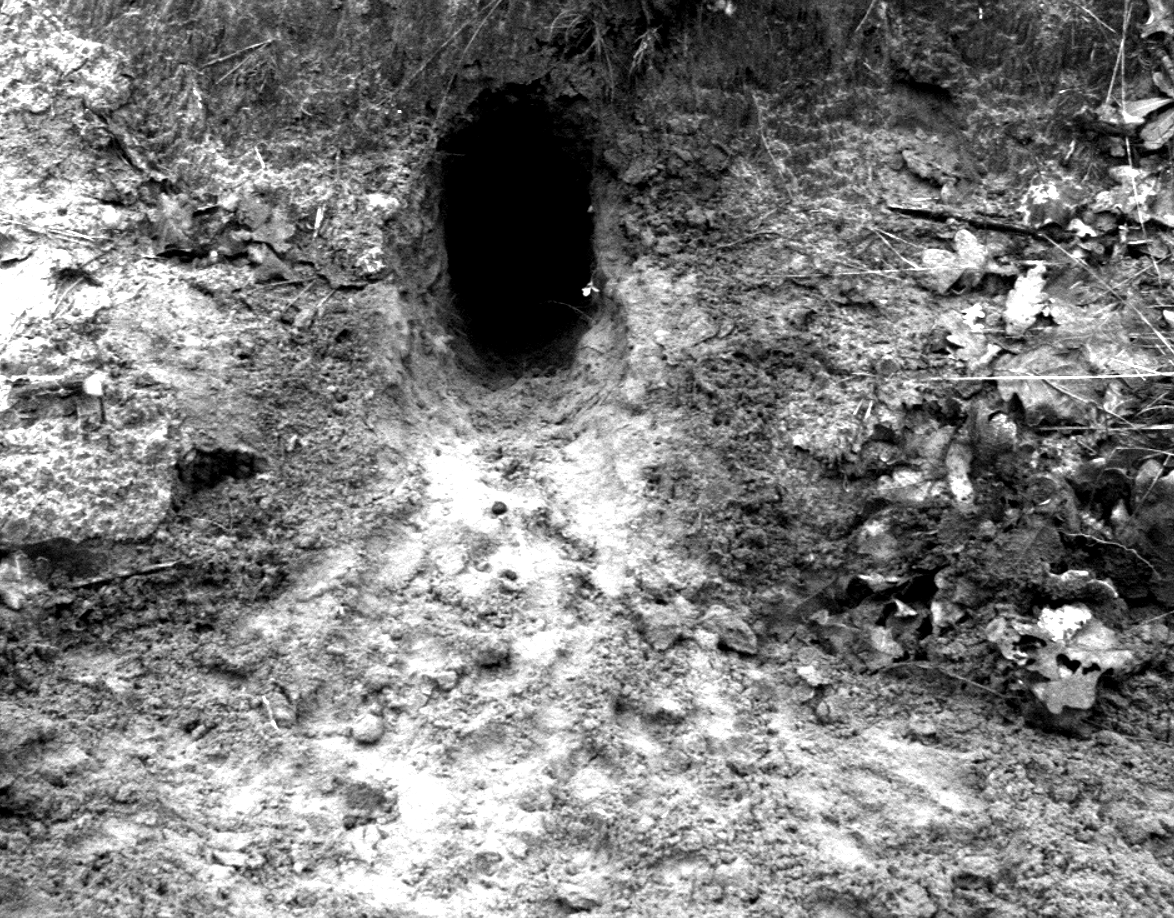

Supplement: SI 4 — (ZIP) [file pcbi.1006535.s014.zip › SI_4/natural_images/img101_VH.tiff]
