# Supplementary material for: Visual physiology of the layer 4 cortical circuit in silico
Source: PLoS Comput Biol. 2018 Nov 12;14(11):e1006535. doi: 10.1371/journal.pcbi.1006535 (PMC6258373; doi:10.1371/journal.pcbi.1006535)
Supplement: S1 Table — The ranges of parameters from which values are randomly selected for each instantiated filter are shown. Properties of the filter responses to visual stimuli are indicated for mean and standard deviation based on spike train responses from 10 trials for gratings and 20 trials for all other stimuli. Orientation selectivity index (OSI) and direction selectivity index (DSI) are computed separately for the F0 and F1 components of the responses to gratings. (DOCX) [file pcbi.1006535.s009.docx]

| LGN filter type / Response property | Transient ON | Transient OFF | Transient ON/OFF |
| --- | --- | --- | --- |
| **Parameters** |  |  |  |
| $R_{0}$ (Hz) | 4.0 - 5.0 | 4.0 - 5.0 | 4.0 - 5.0 |
| $k$ (ms^-1^) | 0.0375 - 0.0385 | 0.0375 - 0.0385 | 0.0375 - 0.0385 |
| $\sigma_{C}$ (degrees) | 2.0 - 3 .0 | 2.0 - 3 .0 | 2.0 - 3 .0 |
| Range for offset between ON and OFF centers (degrees) | - | - | 3.5 - 5.0 |
| **Response Properties** |  |  |  |
| Spontaneous rate (Hz) | 4.5+/-0.3 | 4.5+/-0.3 | 4.5+/-0.3 |
| Gratings: max F0 (Hz) | 12+/-1 | 11+/-1 | 12+/-7 |
| Gratings: max F1 (Hz) | 18+/-2 | 18+/-2 | 13+/-11 |
| Gratings: preferred SF (cpd) | 0.05+/-0.01 | 0.05+/-0.01 | 0.07+/-0.03 |
| Gratings: preferred TF (Hz) | 4.0+/-0.1 | 4.0+/-0.1 | 4.0+/-0.4 |
| Gratings: OSI for F0 | 0.07+/-0.03 | 0.07+/-0.03 | 0.08+/-0.04 |
| Gratings: DSI for F0 | 0.07+/-0.04 | 0.07+/-0.04 | 0.07+/-0.04 |
| Gratings: OSI for F1 | 0.07+/-0.03 | 0.07+/-0.03 | 0.6+/-0.2 |
| Gratings: DSI for F1 | 0.07+/-0.04 | 0.07+/-0.04 | 0.07+/-0.05 |
| Natural images:  time to peak (ms) | 100+/-50 | 100+/-60 | 110+/-80 |
| Full-field flash:  time to peak (ms) | 101+/-13 | 101+/-14 | 102+/-41 |
