# Supplementary material for: Visual physiology of the layer 4 cortical circuit in silico
Source: PLoS Comput Biol. 2018 Nov 12;14(11):e1006535. doi: 10.1371/journal.pcbi.1006535 (PMC6258373; doi:10.1371/journal.pcbi.1006535)
Supplement: S2 Table — The simulated systems (of the LL, RL, LR, and RR types) are indicated together with the path name in the file repository. The five types of visual stimuli, as well as spontaneous activity (“Spont.”) are listed in columns. If simulations of a particular type were performed for a given system, they are indicated together with the corresponding file name suffix and the number of trials. (DOCX) [file pcbi.1006535.s010.docx]

| **System** | **Spont.** | **Gratings (SF = 0.05 cpd)** | **Full-field Flashes** | **Natural Images** | **Natural Movies** | **Moving Bars** |
| --- | --- | --- | --- | --- | --- | --- |
| **LL1**  **(simualtions_ll1/)** | spont: 20 trials  **Inputs from bkg only;**  **…tw_only…** 10 trials each | **Contrast = 80%; TF = 2 Hz; 8 directions**  g(7, 37, 67, 97, 127, 157, 187, 217): 10 trials each  **Contrast = 80%; TF = 4 Hz; 8 directions**  g(8, 38, 68, 98, 128, 158, 188, 218): 10 trials each  **Contrast = 80%; TF = 8 Hz; 8 directions**  g(9, 39, 69, 99, 129, 159, 189, 219): 10 trials each  **No recurrent connections; inputs from LGN only;**  **Contrast = 80%; TF = 4 Hz; 8 directions**  g(8, 38, 68, 98, 128, 158, 188, 218)_ …_LGN_only_no_con: 10 trials each  **No recurrent connections; inputs from LGN only;**  **Contrast = 80%; TF = 2 Hz; 8 directions**  g(7, 37, 67, 97, 127, 157, 187, 217)_ …_LGN_only_no_con: 10 trials each  **OPTOGENETIC silencing of LGN,**  **Contrast = 80%; TF = 4 Hz; 1 direction**  g8_..._stop_1s_...: 10 trials | flash_1: 10 trials |  |  | Bbar_v50pixps_hor: 10 trials  Bbar_v50pixps_vert: 10 trials  Wbar_v50pixps_hor: 10 trials  Wbar_v50pixps_vert: 10 trials |
| **LL2**  **(simualtions_ll2/)** | spont: 20 trials  **Inputs from bkg only;**  **…tw_only…** 10 trials each | **Contrast = 80%; TF = 2 Hz; 8 directions**  g(7, 37, 67, 97, 127, 157, 187, 217): 10 trials each  **Contrast = 80%; TF = 4 Hz; 8 directions**  g(8, 38, 68, 98, 128, 158, 188, 218): 10 trials each  **Contrast = 80%; TF = 8 Hz; 8 directions**  g(9, 39, 69, 99, 129, 159, 189, 219): 10 trials each  **Contrast = 30%; TF = 4 Hz; 8 directions**  g(8, 38, 68, 98, 128, 158, 188, 218)_..._ctr30: 10 trials each  **Contrast = 10%; TF = 4 Hz; 8 directions**  g(8, 38, 68, 98, 128, 158, 188, 218)_..._ctr10: 10 trials each  **No recurrent connections; inputs from LGN only;**  **Contrast = 80%; TF = 4 Hz; 8 directions**  g(8, 38, 68, 98, 128, 158, 188, 218)_ …_LGN_only_no_con: 10 trials each  **No recurrent connections; inputs from LGN only;**  **Contrast = 80%; TF = 2 Hz; 8 directions**  g(7, 37, 67, 97, 127, 157, 187, 217)_ …_LGN_only_no_con: 10 trials each  **No recurrent connections; inputs from LGN only;**  **Contrast = 30%; TF = 4 Hz; 8 directions**  g(8, 38, 68, 98, 128, 158, 188, 218)_..._ctr30…_LGN_only_no_con: 10 trials each  **No recurrent connections; inputs from LGN only;**  **Contrast = 10%; TF = 4 Hz; 8 directions**  g(8, 38, 68, 98, 128, 158, 188, 218)_..._ctr10…_LGN_only_no_con: 10 trials each  **Short tests; Contrast = 80%; TF = 4 Hz**  g8_8_sd278_test500ms: 1 trial  g8_8_sd278_test500ms_LGN_only_no_con: 1 trial  g8_8_sd278_test500ms_no_con: 1 trial  **OPTOGENETIC suppression of 20% Scnn1a, including LIF,**  **Injection current -30 pA,**  **Contrast = 80%; TF = 2 Hz; 1 direction**  g7_..._Sup_Scnn1a_30_LIF_fraction20: 10 trials  **OPTOGENETIC suppression of 20% Scnn1a, including LIF,**  **Injection current -50 pA,**  **Contrast = 80%; TF = 2 Hz; 1 direction**  g7_..._Sup_Scnn1a_50_LIF_fraction20: 10 trials  **OPTOGENETIC suppression of 20% Scnn1a, including LIF,**  **Injection current -100 pA,**  **Contrast = 80%; TF = 2 Hz; 1 direction**  g7_..._Sup_Scnn1a_100_LIF_fraction20: 10 trials  **OPTOGENETIC suppression of 20% Scnn1a, including LIF,**  **Injection current -150 pA,**  **Contrast = 80%; TF = 2 Hz; 1 direction**  g7_..._Sup_Scnn1a_150_LIF_fraction20: 10 trials  **OPTOGENETIC suppression of 50% Scnn1a, including LIF,**  **Injection current -30 pA,**  **Contrast = 80%; TF = 2 Hz; 1 direction**  g7_..._Sup_Scnn1a_30_LIF_fraction50: 10 trials  **OPTOGENETIC suppression of 50% Scnn1a, including LIF,**  **Injection current -50 pA,**  **Contrast = 80%; TF = 2 Hz; 1 direction**  g7_..._Sup_Scnn1a_50_LIF_fraction50: 10 trials  **OPTOGENETIC suppression of 50% Scnn1a, including LIF,**  **Injection current -100 pA,**  **Contrast = 80%; TF = 2 Hz; 1 direction**  g7_..._Sup_Scnn1a_100_LIF_fraction50: 10 trials  **OPTOGENETIC suppression of 50% Scnn1a, including LIF,**  **Injection current -150 pA,**  **Contrast = 80%; TF = 2 Hz; 1 direction**  g7_..._Sup_Scnn1a_150_LIF_fraction50: 10 trials  **OPTOGENETIC suppression of 100% Scnn1a, including LIF,**  **Injection current -30 pA,**  **Contrast = 80%; TF = 2 Hz; 1 direction**  g7_..._Sup_Scnn1a_30_LIF: 10 trials  **OPTOGENETIC suppression of 100% Scnn1a, including LIF,**  **Injection current -50 pA,**  **Contrast = 80%; TF = 2 Hz; 1 direction**  g7_..._Sup_Scnn1a_50_LIF: 10 trials  **OPTOGENETIC suppression of 100% Scnn1a, including LIF,**  **Injection current -100 pA,**  **Contrast = 80%; TF = 2 Hz; 1 direction**  g7_..._Sup_Scnn1a_100_LIF: 10 trials  **OPTOGENETIC suppression of 100% Scnn1a, including LIF,**  **Injection current -150 pA,**  **Contrast = 80%; TF = 2 Hz; 1 direction**  g7_..._Sup_Scnn1a_150_LIF: 10 trials  **OPTOGENETIC suppression of 100% Scnn1a, including LIF,**  **Injection current -30 pA,**  **Contrast = 80%; TF = 4 Hz; 1 direction**  g8_..._Sup_Scnn1a_30_LIF: 10 trials  **OPTOGENETIC suppression of 100% Scnn1a, including LIF,**  **Injection current -50 pA,**  **Contrast = 80%; TF = 4 Hz; 1 direction**  g8_..._Sup_Scnn1a_50_LIF: 10 trials  **OPTOGENETIC suppression of 100% Scnn1a, including LIF,**  **Injection current -100 pA,**  **Contrast = 80%; TF = 4 Hz; 1 direction**  g8_..._Sup_Scnn1a_100_LIF: 10 trials  **OPTOGENETIC suppression of 100% Scnn1a, including LIF,**  **Injection current -150 pA,**  **Contrast = 80%; TF = 4 Hz; 1 direction**  g8_..._Sup_Scnn1a_150_LIF: 10 trials  **OPTOGENETIC suppression of 100% Scnn1a, including LIF,**  **Injection current -150 pA,**  **Contrast = 80%; TF = 4 Hz; 1 direction**  g8_..._Sup_Scnn1a_150_LIF: 10 trials  **OPTOGENETIC suppression of 100% Scnn1a,**  **Injection current -50 pA,**  **Contrast = 80%; TF = 4 Hz; 1 direction**  g8_..._sup5: 10 trials  **OPTOGENETIC suppression of 100% Rorb,**  **Injection current -50 pA,**  **Contrast = 80%; TF = 4 Hz; 1 direction**  g8_..._sup1: 10 trials  **OPTOGENETIC suppression of 100% Nr5a1,**  **Injection current -70 pA,**  **Contrast = 80%; TF = 4 Hz; 1 direction**  g8_..._sup3: 10 trials  **OPTOGENETIC suppression of 100% PV1,**  **Injection current -70 pA,**  **Contrast = 80%; TF = 4 Hz; 1 direction**  g8_..._sup4: 10 trials  **OPTOGENETIC suppression of 100% PV2,**  **Injection current -50 pA,**  **Contrast = 80%; TF = 4 Hz; 1 direction**  g8_..._sup2: 10 trials  **OPTOGENETIC excitation of 100% Scnn1a,**  **Injection current 160 pA,**  **Contrast = 80%; TF = 4 Hz; 1 direction**  g8_..._test5: 10 trials  **OPTOGENETIC excitation of 100% Rorb,**  **Injection current 200 pA,**  **Contrast = 80%; TF = 4 Hz; 1 direction**  g8_..._test1: 10 trials  **OPTOGENETIC excitation of 100% Nr5a1,**  **Injection current 230 pA,**  **Contrast = 80%; TF = 4 Hz; 1 direction**  g8_..._test3: 10 trials  **OPTOGENETIC excitation of 100% PV1,**  **Injection current 340 pA,**  **Contrast = 80%; TF = 4 Hz; 1 direction**  g8_..._test4: 10 trials  **OPTOGENETIC excitation of 100% PV2,**  **Injection current 210 pA,**  **Contrast = 80%; TF = 4 Hz; 1 direction**  g8_..._test2: 10 trials  **OPTOGENETIC silencing of LGN,**  **Contrast = 80%; TF = 4 Hz; 1 direction**  g8_..._stop_1s_...: 10 trials  **LGN inputs removed from V-clamped cells,**  **Contrast = 80%; TF = 2 Hz; 8 directions**  g(7, 37, 67, 97, 127, 157, 187, 217)_ ..._remove_inputs_from_SEClamp_cells:  1 trial each  **V-clamping at 0 mV (measure inh. Currents),**  **Contrast = 80%; TF = 2 Hz; 8 directions**  g(7, 37, 67, 97, 127, 157, 187, 217)  _..._SEClamp_e0:  10 trials each | flash_1: 10 trials  flash_2: 10 trials | imseq_(0-99): 1 trial each | TouchOfEvil_frames_1530_to_1680: 10 trials  TouchOfEvil_frames_3600_to_3750: 10 trials  TouchOfEvil_frames_5550_to_5700: 10 trials  **Scrambled:**  TouchOfEvil_frames_3600_to_3750_scrbl_t:  10 trials  TouchOfEvil_frames_3600_to_3750_scrbl_xy:  10 trials | Bbar_v50pixps_hor:  10 trials  Bbar_v50pixps_vert: 10 trials  Wbar_v50pixps_hor: 10 trials  Wbar_v50pixps_vert: 10 trials |
| **LL3**  **(simualtions_ll3/)** | spont: 20 trials  **Inputs from bkg only;**  **…tw_only…** 10 trials each | **Contrast = 80%; TF = 2 Hz; 8 directions**  g(37, 67, 97, 127, 157, 187, 217): 10 trials each  **Contrast = 80%; TF = 4 Hz; 8 directions**  g(8, 38, 68, 98, 128, 158, 188, 218): 10 trials each  **Contrast = 80%; TF = 8 Hz; 8 directions**  g(9, 39, 69, 99, 129, 159, 189, 219): 10 trials each  **No recurrent connections; inputs from LGN only;**  **Contrast = 80%; TF = 2 Hz; 8 directions**  g(7, 37, 67, 97, 127, 157, 187, 217)_ …_LGN_only_no_con: 10 trials each  **No recurrent connections; inputs from LGN only;**  **Contrast = 80%; TF = 4 Hz; 8 directions**  g(8, 38, 68, 98, 128, 158, 188, 218)_ …_LGN_only_no_con: 10 trials each  **OPTOGENETIC silencing of LGN,**  **Contrast = 80%; TF = 4 Hz; 1 direction**  g8_..._stop_1s_...: 10 trials | flash_1: 10 trials  flash_2: 10 trials | imseq_(0-99): 1 trial each | TouchOfEvil_frames_1530_to_1680: 10 trials  TouchOfEvil_frames_3600_to_3750: 10 trials  TouchOfEvil_frames_5550_to_5700: 10 trials | Bbar_v50pixps_hor: 10 trials  Bbar_v50pixps_vert: 10 trials  Wbar_v50pixps_hor: 10 trials  Wbar_v50pixps_vert: 10 trials |
| **RL1**  **(simualtions_rl1/)** | spont: 20 trials | **Contrast = 80%; TF = 4 Hz; 8 directions**  g(8, 38, 68, 98, 128, 158, 188, 218): 10 trials each | flash_1: 10 trials |  |  |  |
| **RL2**  **(simualtions_rl2/)** | spont: 20 trials | **Contrast = 80%; TF = 4 Hz; 8 directions**  g(8, 38, 68, 98, 128, 158, 188, 218): 10 trials each | flash_1: 10 trials  flash_2: 10 trials |  | TouchOfEvil_frames_1530_to_1680: 10 trials  TouchOfEvil_frames_3600_to_3750: 10 trials  TouchOfEvil_frames_5550_to_5700: 10 trials |  |
| **RL3**  **(simualtions_rl3/)** | spont: 20 trials | **Contrast = 80%; TF = 4 Hz; 8 directions**  g(8, 38, 68, 98, 128, 158, 188, 218): 10 trials each | flash_1: 10 trials  flash_2: 10 trials |  | TouchOfEvil_frames_1530_to_1680: 10 trials  TouchOfEvil_frames_3600_to_3750: 10 trials  TouchOfEvil_frames_5550_to_5700: 10 trials |  |
| **LR1**  **(simualtions_lr1/)** | spont: 20 trials | **Contrast = 80%; TF = 4 Hz; 8 directions**  g(8, 38, 68, 98, 128, 158, 188, 218): 10 trials each | flash_1: 10 trials |  |  |  |
| **LR2**  **(simualtions_lr2/)** | spont: 20 trials | **Contrast = 80%; TF = 4 Hz; 8 directions**  g(8, 38, 68, 98, 128, 158, 188, 218): 10 trials each | flash_1: 10 trials  flash_2: 10 trials |  | TouchOfEvil_frames_1530_to_1680: 10 trials  TouchOfEvil_frames_3600_to_3750: 10 trials  TouchOfEvil_frames_5550_to_5700: 10 trials |  |
| **LR3**  **(simualtions_lr3/)** | spont: 20 trials | **Contrast = 80%; TF = 4 Hz; 8 directions**  g(8, 38, 68, 98, 128, 158, 188, 218): 10 trials each | flash_1: 10 trials  flash_2: 10 trials |  | TouchOfEvil_frames_1530_to_1680: 10 trials  TouchOfEvil_frames_3600_to_3750: 10 trials  TouchOfEvil_frames_5550_to_5700: 10 trials |  |
| **RR1**  **(simualtions_rr1/)** | spont: 20 trials | **Contrast = 80%; TF = 4 Hz; 8 directions**  g(8, 38, 68, 98, 128, 158, 188, 218): 10 trials each | flash_1: 10 trials |  |  |  |
| **RR2**  **(simualtions_rr2/)** | spont: 20 trials | **Contrast = 80%; TF = 4 Hz; 8 directions**  g(8, 38, 68, 98, 128, 158, 188, 218): 10 trials each | flash_1: 10 trials  flash_2: 10 trials | imseq_(0-99): 1 trial each | TouchOfEvil_frames_1530_to_1680: 10 trials  TouchOfEvil_frames_3600_to_3750: 10 trials  TouchOfEvil_frames_5550_to_5700: 10 trials |  |
| **RR3**  **(simualtions_rr3/)** | spont: 20 trials | **Contrast = 80%; TF = 4 Hz; 8 directions**  g(8, 38, 68, 98, 128, 158, 188, 218): 10 trials each | flash_1: 10 trials  flash_2: 10 trials | imseq_(0-99): 1 trial each | TouchOfEvil_frames_1530_to_1680: 10 trials  TouchOfEvil_frames_3600_to_3750: 10 trials  TouchOfEvil_frames_5550_to_5700: 10 trials |  |
